# Optimized Protocol for High-Quality RNA Extraction from Grape Berry Skins Using Sorbitol Pre-Wash

**DOI:** 10.3390/plants14070988

**Published:** 2025-03-21

**Authors:** Annalisa Prencipe, Antonella Salerno, Margherita D’Amico, Antonio Domenico Marsico, Mario Ventura, Riccardo Velasco, Maria Francesca Cardone, Carlo Bergamini, Flavia Angela Maria Maggiolini

**Affiliations:** 1 Department of Biosciences, Biotechnology and Environment, University of Bari “Aldo Moro”, 70125 Bari, Italy; 2 Council for Agricultural Research and Economics—Research Center Viticulture and Enology (CREA-VE), Via Casamassima 148, 70010 Turi, Italy

**Keywords:** *Vitis vinifera*, RNA, sorbitol, extraction protocol

## Abstract

Extracting high-quality RNA from grape (*Vitis Vinifera*) berry skins is challenging due to their high levels of polysaccharides, phenolic compounds, sugars, and organic acids, which can negatively impact RNA purity and yield. Indeed, polyphenols can bind to RNA, polysaccharides may co-precipitate, and sugars and organic acids can interfere with the pH and ionic properties of the extraction buffer. Commercial kits offer a quick extraction method but are often ineffective for grape berry skins. Similarly, protocols that work well for other vegetal tissues are also inefficient and time-consuming for this tissue. To overcome these limitations, we optimized the RNA isolation by adding a sorbitol pre-wash step to both a non-commercial protocol and a commercial kit. Our results show that it significantly improves the RNA yield and quality from grape berry skins, increasing the RNA purity and integrity, as evidenced by higher RIN (RNA Integrity Number) values and better Qubit and Nanodrop measurements. The strategy’s efficacy was further validated through RNA sequencing, yielding high-quality reads with low error rates, suitable for gene expression studies. Thus, incorporating a sorbitol pre-wash step improves the RNA yield and quality from grape berry skins making it suitable for high-throughput sequencing, and provides a reliable tool for advancing grapevine research.

## 1. Introduction

High-quality RNA, free from DNA contamination, is essential for various plant molecular biology techniques including RT-PCR, qPCR, cDNA library construction, northern blots, in situ hybridization, and gene expression analysis. The increasing use of Next-Generation Sequencing (NGS) [1,2,3] has further enhanced the need for pure, intact, and high-yield RNA, as sequencing accuracy depends on RNA integrity (measured by the RIN), purity (A260/280 and A260/230 ratios), and yield [4,5].

RNA extraction protocols were initially optimized for model herbaceous plants like Arabidopsis [6], but applying these methods to different plant species remains challenging due to the diversity of plant biochemical profiles in terms of both primary and secondary metabolites [5,7]. The quality of RNA extraction is influenced by multiple factors, including the plant growth stage, tissue type, age, physiological status, and growing conditions [5,8,9]. Therefore, protocols must be optimized for specific species and tissues to ensure high yields, minimal degradation, and contaminant removal [10]. Woody plants are especially difficult because they contain high levels of polysaccharides and polyphenols, which interfere with RNA isolation by co-precipitating with RNA or irreversibly binding to nucleic acids and proteins. These contaminants negatively impact enzymatic reactions in downstream applications, making high-quality RNA extraction more difficult [5]. This is why immature tissues like seedling leaves are often preferred due to their lower polysaccharide and polyphenol content [11,12]. Various approaches have been developed to mitigate these issues, including high-salt CTAB (cetyltrimethylammonium bromide)/PVP (polyvinylpyrrolidone) and SDS (sodium dodecyl sulfate)-based extraction methods, commonly used to remove polysaccharide contamination. However, not all plant species respond well to these approaches, particularly those rich in secondary metabolites that can interfere with CTAB/PVP and SDS efficiency [13]. Additionally, many of these methods are time-consuming, require hazardous chemicals like phenol and chloroform [14,15,16], and have limited throughput, making them less practical for large-scale transcriptomic studies. TRIZOL-based RNA extraction, while widely used, also presents challenges, particularly in high-throughput applications, due to the presence of organic contaminants in the final extracts [7]. Grapevines (*Vitis* spp.) are one of the most extensively cultivated woody plants worldwide, used for both wine and fresh fruit production. While several protocols have been developed for RNA extraction from different grapevine tissues, many are technically complex, time-consuming, or yield inconsistent results. Recent studies, such as Song et al. [17] and Reid et al. [18], have demonstrated high RNA yields from grape tissues using optimized protocols. However, these methods often require specialized kits and additional steps, or their performance varies across different grape tissues. Commercial kits, such as the Sigma Spectrum Plant Total RNA Kit, have been reported as effective, but their high cost and limited availability—with some kits discontinued or unavailable in certain regions, including Italy—make them impractical for many laboratories. Additionally, many widely used protocols struggle to extract high-quality RNA from grape berries, which are rich in tannins, polysaccharides, and other interfering compounds [19,20,21]. These challenges highlight the need for an improved cost-effective, reproducible, and scalable RNA extraction method.

In this study, we optimized an RNA extraction protocol by incorporating a sorbitol pre-wash step into both a non-commercial extraction protocol and a commercial kit-based method. Sorbitol has been previously used in DNA extraction to remove interfering compounds without precipitating nucleic acids [22,23,24,25], but very few studies have investigated its impact on RNA extraction [26,27]. Unlike ethanol, sorbitol stabilizes cell membranes and selectively removes polyphenols and polysaccharides without precipitating RNA, making it particularly suitable for grape berry skins [26,28]. Furthermore, to the best of our knowledge, sorbitol has never been tested in grape samples for RNA extraction. Our findings indicate that the effectiveness of the sorbitol treatment is influenced by the type of tissue being processed and it can significantly enhance RNA yield and purity, particularly in polyphenol and anthocyanin-rich tissues.

## 2. Results

### 2.1. Enhancing RNA Extraction from Grape Berry Skins: The Impact of a Sorbitol Pre-Wash on Yield, Purity, and Integrity

**Figure 1:**
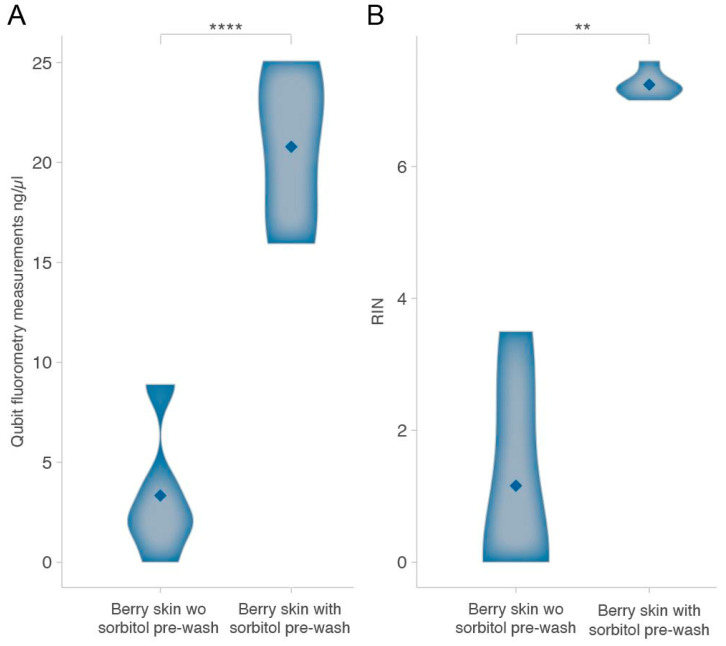
Yield and quality of RNA extracted from grape berry skins using the commercial kit. (**A**). Violin plot showing Qubit concentration measurements of five grape berry skins treated with a sorbitol pre-wash compared to untreated skins. (**B**). Violin plot showing the RIN values of five grape berry skins treated with a sorbitol pre-wash compared to untreated skins. Asterisks indicate significant differences (**** *p* < 0.0001 and ** *p* < 0.01) according to the Student’s *t*-test. wo sorbitol = without sorbitol.

RNA extraction from grape berry skins was performed using both a non-commercial protocol and a commercial kit-based method, with the RNA quality assessed through Qubit, Nanodrop, and Bioanalyzer analyses. Without a sorbitol pre-wash, both methods resulted in poor RNA quality, characterized by low yields, high contamination levels, and extensive degradation. Indeed, Qubit fluorometry indicated low RNA concentrations, and nanodrop spectrophotometry revealed significant contamination, with 260/280 ratios well below the optimal 2.0 range (at least for the non-commercial kit), indicating protein contamination, and low 260/230 ratios suggesting the presence of polysaccharides and phenolic compounds. The Bioanalyzer analysis showed almost no RNA recovery, with RIN values too low to be determined, making the RNA unsuitable for downstream applications (Supplementary Figure S1A,B). To improve RNA yield and integrity, a sorbitol pre-wash step was introduced (Supplementary Figure S2 and methods). This additional step significantly enhanced RNA quality, particularly reflected in the RIN values, which provide a comprehensive measure of RNA integrity by accounting for degradation and contamination. The impact of the sorbitol pre-wash was assessed across five samples for each method (commercial and non-commercial kits, Supplementary Figure S1C,D). The non-commercial extraction method, when performed without sorbitol, resulted in an average RNA yield of 2.0 ng/µL, a 260/280 ratio of 0.74, and RIN values too low to be assessed. With the sorbitol pre-wash, the RNA yield increased to 32.85 ng/µL, while the 260/280 ratio improved to 1.8. The Bioanalyzer analysis showed a marked improvement in RNA integrity, with RIN values exceeding 7.0, confirming the effectiveness of the pre-wash in removing contaminants and preserving the RNA quality (Supplementary Figure S1D). Using the commercial kit without sorbitol, it yielded 3.3 ng/µL of RNA, with a 260/280 ratio ranging from 1.88 to 2.5. The Bioanalyzer analysis revealed extensive RNA degradation, with an average RIN of 1.2, making the RNA unsuitable for RNA-seq applications. With the sorbitol pre-wash, the RNA yield increased six-fold to 20.8 ng/µL, and while the 260/230 ratio remained below the optimal 1.8 threshold, it showed noticeable improvement (Supplementary Table S1). The most significant change was observed in RNA integrity, with the RIN values rising from 1.2 to 7.2, demonstrating a substantial increase in RNA suitability for downstream applications (Figure 1A,B and Supplementary Table S1). Finally, when comparing the two protocols after the implementation of the sorbitol pre-wash, the non-commercial method outperformed the kit-based approach in terms of RNA yield (32.85 ng/µL vs. 20.8 ng/µL) while achieving comparable improvements in purity and RNA integrity.

Considering that the non-commercial protocol is more time-consuming (three days of work, see methods), we further validated our findings by systematically testing the suitability of the proposed method by applying it to an extended sample set (n = 38) using the commercial kit. The Qubit measurements from this larger dataset confirmed consistent RNA yields (19.3 ng/µL), with a 260/280 ratio of 1.9 and a mean RIN of 6.7 (Supplementary Table S2). These results collectively highlight the crucial role of the sorbitol pre-wash in improving the RNA yield and purity, with a particularly strong effect on RIN values.

### 2.2. Validation of RNA Extraction Protocol Through High-Throughput Analysis

To further demonstrate the efficacy of this extraction protocol, RNA samples extracted from grape berries were sent to the Novogene company for RNA sequencing (RNA-seq). The cDNA libraries were prepared, and the mRNAs were sequenced using the Illumina NovaSeq platform, which utilizes a paired-end 150 bp sequencing strategy. This high-throughput sequencing method ensures comprehensive coverage and accuracy in the analysis of gene expression. A total of 33 cDNA libraries were constructed and sequenced, yielding an average of 56.7 million raw reads per library. Quality control measures ensured that 95.7% of these reads were classified as clean reads, with a low base error rate of 0.01. The sequencing data underwent thorough analysis to calculate key statistics, including the total number of raw reads and clean reads, GC content (%), Q20 (%), and Q30 (%). These statistics were assessed for all 33 samples and are summarized in Supplementary Table S3. The Q20 scores ranged from 96.66% to 97.24% and the Q30 scores ranged from 90.40% to 93.52%. These results validated the RNA extraction method as highly effective for generating high-quality RNA, which is essential for intricate gene expression studies. The high percentage of clean reads (Q20 and Q30 scores) and low error rates (0.01) confirmed the method’s robustness and suitability for producing high-quality data. Moreover, we validated the effectiveness of our RNA extraction method in producing RNA that can be used in RNA-seq applications by analyzing the gene expression levels from our dataset. The actin housekeeping gene (Vitvi04g01613) exhibited consistently high expression across all the replicates, with Fragments Per Kilobase of transcript sequence per Millions base pairs sequenced (FPKM) values ranging from 792.93 to 876.79, confirming the integrity and reliability of the extracted RNA. Importantly, our method enabled the detection of a substantial number of genes that are expressed at low levels. Specifically, 1353 genes exhibited expression levels between 0 and 1 in all the replicates. For instance, Vitvi03g00264, Vitvi03g00284, and Vitvi01g00427 exhibited very low FPKM values (as low as 0.005) yet remained detectable in our dataset (Supplementary Table S4).

**Figure 2:**
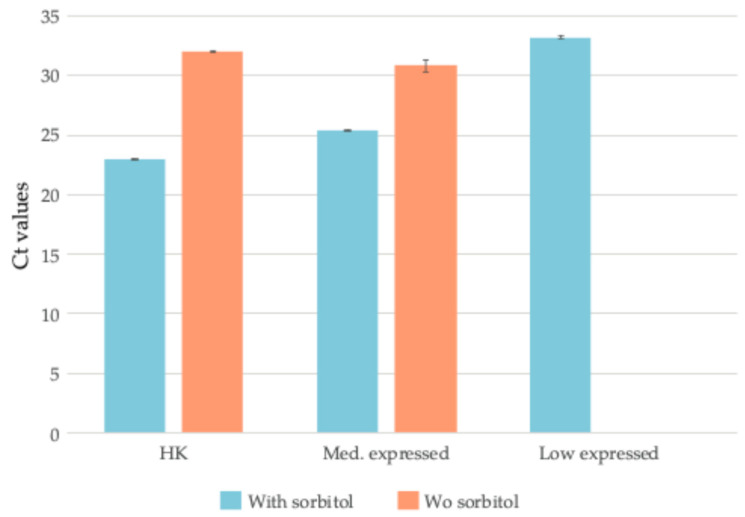
qPCR analysis of RNA extracted using the commercial kit with and without the sorbitol pre-wash. The figure shows the Ct values for three genes—Vitvi04g01613 (housekeeping gene, high expression), Vitvi18g02133 (medium expression), and Vitvi03g00231 (low expression)—measured using 20 ng of cDNA obtained from RNA extracted with and without the sorbitol pre-wash.

To further evaluate the method, we performed qPCR on cDNA extracted from the same sample with and without sorbitol treatment. Initially, we assessed two housekeeping genes, actin (ACTN, Vitvi18g02133) and glyceraldehyde-3-phosphate dehydrogenase (GAPC, Vitvi17g01598), using different cDNA input amounts (20 ng, 30 ng, 50 ng, and 100 ng). The Ct values for both genes were consistently lower in the sorbitol-treated samples compared to those without sorbitol, indicating a higher RNA quality and quantity. Specifically, for actin, the Ct values ranged from 22.57 (20 ng) to 19.60 (100 ng) with sorbitol, whereas without sorbitol, they were significantly higher, ranging from 32.30 (20 ng) to 30.02 (100 ng) (Supplementary Figure S3). Similarly, for GAPC, the Ct values ranged from 19.43 (20 ng) to 17.32 (100 ng) with sorbitol, while in the untreated samples, they were much higher, ranging from 30.86 (20 ng) to 29.06 (100 ng) (Supplementary Figure S3). Thus, we selected three genes based on their expression levels (FPKM) from our RNA-seq data: Vitvi04g01613 (housekeeping gene; FPKM: 876.79), Vitvi18g02133 (medium expression; FPKM: 113.76), and Vitvi03g00231 (low expression; FPKM: 1.22). Using 20 ng of cDNA, the qPCR analysis showed that the Ct values for all three genes were consistently lower in sorbitol-treated samples compared to untreated ones (Figure 2, Supplementary Figure S4A–C). Notably, for the gene with low expression (Vitvi03g00231), the melting curve analysis (Supplementary Figure S4C) indicated that the amplification in the untreated sample was non-specific, confirming that the gene is undetectable without the sorbitol pre-wash step.

### 2.3. Differential Impact of Sorbitol Pre-Wash on RNA Extraction from Different Grape Tissues

**Figure 3:**
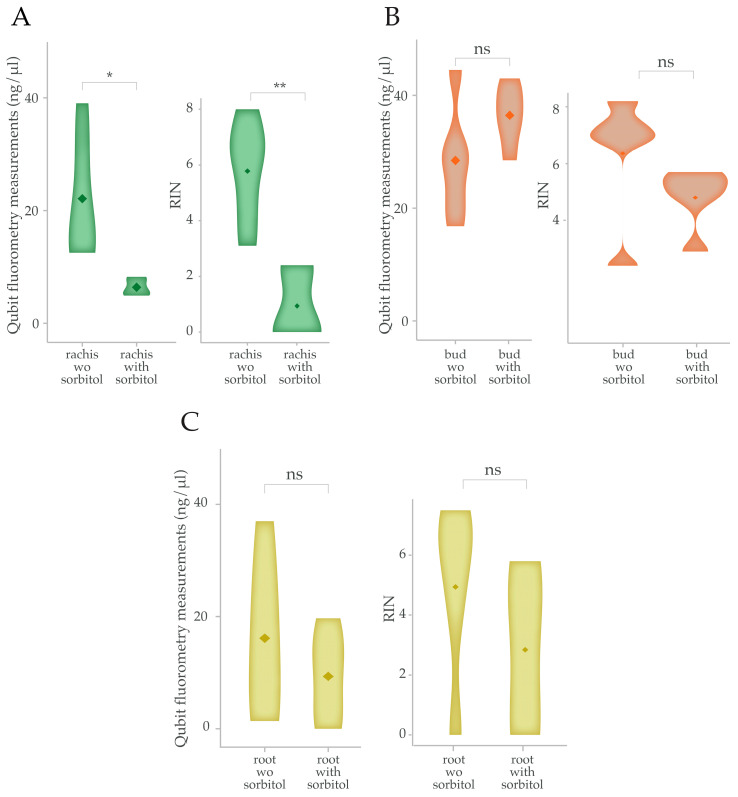
Yield and quality of RNA extracted from other grape tissues using the commercial kit. Violin plots showing Qubit concentration measurements and RIN values of RNA from grape rachis (**A**), buds (**B**), and roots (**C**) with the sorbitol pre-wash compared to those without the pre-wash. Asterisks indicate significant differences (** *p* < 0.01; * *p* < 0.05; ns *p* > 0.05) according to the Student’s *t*-test. wo sorbitol = without sorbitol.

To evaluate the applicability of the extraction protocol with the sorbitol pre-wash across various grape tissues, we extended our analysis to grape rachises, buds, and roots. The effect of the sorbitol pre-wash and the following RNA extraction with the kit protocol were assess based on the RNA yield and quality from these tissues, comparing the results with those of the same samples without the sorbitol pre-wash. In contrast to the results obtained from the berry skins, the rachis samples gave better RNA extraction results without the sorbitol pre-wash treatment. The RNA yields for the rachis samples without the pre-wash averaged 22.1 ng/µL, while those with the pre-wash averaged 6.4 ng/µL. The RNA integrity followed a similar pattern, with significantly better RIN values without the pre-wash compared to the samples treated with the pre-wash (Figure 3A, Supplementary Table S1, and Table 1).

For grape buds, the RNA yields and RIN showed no significant differences between the two treatment methods (Figure 3B, Supplementary Table S1, and Table 1). Similarly, the grape root samples did not exhibit significant differences in the RNA extraction outcomes between the two methods (Figure 3C, Supplementary Table S1, and Table 1). These findings suggest that while the sorbitol pre-wash step significantly enhances RNA yield and integrity from grape berry skins, its benefits are not universal across all grape tissues.

## 3. Discussion

The extraction of high-quality DNA-free RNA is essential for advances in plant molecular biology, a process that has been significantly enhanced by the advent of NGS techniques. Despite this progress, obtaining quality RNA from a wide range of plant species, particularly from woody plants such as grapevines, remains a challenge. Over the years, numerous commercial RNA extraction kits have been tested on grapevine tissues, with varying levels of success. While some, like the Sigma Spectrum Plant Total RNA Kit, have proven effective for specific grapevine tissues [17], their cost and limited availability pose significant challenges for laboratories working with large sample sets. Indeed, grapevine research often requires RNA extraction from multiple biological replicates across different developmental stages and environmental conditions, making cost-effective solutions highly desirable. Additionally, while certain kits perform well for specific grape tissues, inconsistent performance across different cultivars necessitate the exploration of alternative approaches [10]. Our experience is that most, even from renowned reference companies in the sector, fail to extract high-quality RNA or often fail to recover any RNA. Also, there are notable differences among *Vitis vinifera* varieties in terms of the contents of secondary metabolites [29,30]; these differences may explain why researchers sometimes did not report encountering major difficulties in extracting RNA from some tissues. Currently, the available methods for RNA extraction from grape tissues, particularly berry skins, often yield RNA of insufficient quality for high-throughput sequencing and other molecular analyses and are often complex and time-consuming.

Our results demonstrated that a sorbitol pre-wash effectively enhances RNA extraction from grape berry skins by reducing contaminants and preserving RNA integrity. Unlike ethanol-based washes, sorbitol selectively removes oxidized phenolics and polysaccharides while stabilizing cell membranes, preventing RNA degradation. This is critical since the co-precipitation of contaminants can hinder enzymatic reactions during reverse transcription and RNA sequencing. The efficacy of sorbitol is reflected in the significantly higher RIN values obtained (Figure 1A,B and Supplementary Table S1), consistently above 7, meeting the quality standards for RNA-seq. While A260/230 ratios are commonly used to assess RNA purity, our results confirmed that RNA integrity and sequencing metrics such as Q20/Q30 scores are more reliable indicators of sequencing success. Even samples with A260/230 ratios below 1.8 produced high-quality RNA-seq libraries. Additionally, the sorbitol-treated samples exhibited consistently lower Ct values for different genes with varying expression levels and cDNA inputs (Figure 3, Supplementary Figure S4), and particularly improved the detection of genes expressed at low levels. Without sorbitol, these genes often showed non-specific amplification or remained undetectable (Supplementary Figure S4C). This underscores the importance of sorbitol in minimizing contaminants that could impair qPCR efficiency. The RNA-seq analysis further confirmed the method’s suitability for high-throughput applications, with a high sequencing quality maintained across libraries, Q30 scores exceeding 90%, and 1353 genes with low expression levels (0–1 FPKM) still being detectable (Supplementary Table S4). These findings highlight the sorbitol pre-wash as a valuable refinement for plant RNA extraction, particularly when working with low-expression genes requiring high sensitivity.

However, the benefits of the sorbitol pre-wash were not uniform across all grape tissues, which limits its broader applicability to a range of grapevine tissues. While the sorbitol pre-wash step significantly improved the RNA yield and integrity from grape berry skins, its impact on other tissues such as grape rachises, buds, and roots varied (Table 1, Figure 3). For tissues with inherently lower levels of polyphenols and polysaccharides, such as rachises, the pre-wash step may not be necessary and could potentially lead to lower RNA recovery. This highlights the importance of tailoring RNA extraction methods to specific tissue types, rather than applying a universal approach.

**Figure 4:**
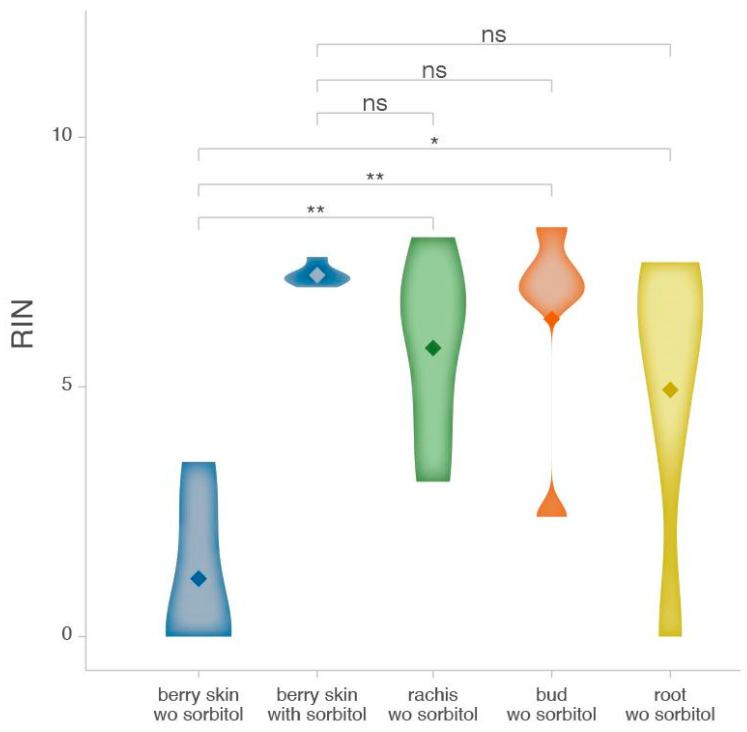
Violin plot showing the impact of the sorbitol pre-wash on the RIN values of RNA extracted from various grape tissues. Comparison of RIN values of sorbitol-treated RNA from berry skins with those of other non treated grape tissues. RNA was extracted using the commercial kit. Asterisks indicate significant differences (** *p* < 0.01; * *p* < 0.05; ns *p* > 0.05) according to the Student’s *t*-test. wo sorbitol = without sorbitol.

Compared to other studies, such as Song et al. [17], which reported RNA yields of 68–300 ng/μL, our protocol using a sorbitol pre-wash did not achieve the same high yield. However, it significantly improves the RNA integrity, as demonstrated by the higher RIN values and suitability for RNA sequencing, emphasizing the need to optimize protocols for tissue-specific challenges rather than focusing solely on yield. Of note, the sorbitol pre-wash also enhanced the consistency and reliability of RNA extraction from berry skins, reducing variability in RIN values (Figure 4), a key factor for high-throughput applications and accurate gene expression analysis. Moreover, the enhancement of both non-commercial and commercial kit-based extraction allows researchers to balance cost-effectiveness and time efficiency: commercial kits are advantageous for processing large numbers of samples due to their reduced hands-on time, while manual protocols remain a viable, less expensive option for smaller-scale experiments despite being more labor-intensive.

In conclusion, our findings align with those of previous studies that have highlighted the challenges of RNA extraction from polyphenol-rich tissues. Our modifications to RNA extraction protocols can address contamination issues in RNA extracted from grape tissues. To our knowledge, this study is the first to demonstrate the application of sorbitol as a pre-wash step specifically for RNA extraction from grape berry skin, highlighting its potential advantages over other conventional approaches. The improved RNA quality obtained with the sorbitol pre-wash protocol has significant implications for molecular and high-throughput applications, as highlighted from the qPCR and RNA-seq validations. Indeed, the gene expression analysis confirmed the reliability of our RNA extraction method, as evidenced by the stable expression of the housekeeping genes and the successful detection of low-abundance transcripts. The ability to obtain consistently high-quality RNA from grape berry skins represents an advancement in plant molecular biology methodologies, offering researchers a cost-effective and reproducible protocol for transcriptomic studies.

## 4. Materials and Methods

### 4.1. Step-by-Step RNA Extraction Protocol with Sorbitol Pre-Wash

#### 4.1.1. Sample Preparation and Sorbitol Pre-Wash

Step 1: Sample preparation

1. Grind 250 mg (for non-commercial procedure) or 100 mg (for kit-based extraction) of frozen grape berry skins or other tissues into fine powder using liquid nitrogen.
2. Divide samples into two groups:
  Without sorbitol pre-wash;
  With sorbitol pre-wash.

Step 2: Sorbitol pre-wash (for selected samples) (20 min)

1. Prepare the pre-wash buffer: 100 mM Tris-HCl (pH 8.0), 0.35 M sorbitol, 5 mM EDTA (pH 8.0), 1% PVP-40, and 1% β-mercaptoethanol (added fresh before use).
2. Add 1.5 mL of pre-wash buffer to macerated tissue.
3. Vortex vigorously and centrifuge at 2500× *g* for 5 min at 4 °C.
4. Discard the supernatant containing polysaccharides and polyphenols.
5. Repeat steps 2 to 4.

#### 4.1.2. RNA Extraction with Non-Commercial Protocol (3 Days) (Modified from Rerie’s Protocol [31])

Day 1: Lysis (work on ice)

1. Add 750 μL of extraction buffer (Tris-HCl 1M/SDS 1%/β-mercaptoethanol 5% + PVP 0.8%) to the sample.
2. Vortex vigorously for 1 min.
3. Add 1 mL of chloroform/isoamyl alcohol (24:1), mix well, and centrifuge.
4. Recover the aqueous phase and precipitate RNA with 80 μL of KCl 2M and then with 330 μL of LiCl 8M.
5. Store at −20 °C overnight.

Day 2: Wash and Precipitation (work on ice)

6. Centrifuge at 12,000× *g* for 20 min at 4 °C.
7. Wash the RNA pellet multiple times with cold 2M LiCl.
8. Resuspend in 1X TE buffer and centrifuge at 12,000× *g* for 10 min at 4 °C.
9. Recover the supernatant and precipitate using 25 μL of 2M potassium acetate (pH 5.5).
10. Incubate 15 min on ice and centrifuge at 12,000× *g* for 15 min at 4 °C.
11. Recover the supernatant and precipitate with 1/10 of the volume of 3M sodium acetate (pH 6) and 2.5 volumes of ice-cold 100% ethanol.
12. Store at −20 °C overnight.

Day 3: Wash and Elution

13. Collect RNA by centrifugation.
14. Wash the pellet with 1 ml of ice-cold 70% ethanol and resuspend in 30 μL of DEPC-treated water.
15. Perform DNase I (Sigma-Aldrich, St. Louis, MO, USA) treatment to remove DNA contamination.

#### 4.1.3. RNA Extraction with Kit Protocol (Kit Plant/Fungi Total RNA Purification Kit from Norgen Biotek, Thorold, ON, Canada) (1 h)

Perform the extraction following the manufacturer instructions using the following steps:

1. Lysate Preparation
  - Transfer the powder to a 2 mL centrifuge tube and add 600 μL of Lysis Buffer C. Vortex vigorously.
  - Incubate at 55 °C for 5 min. Mix the lysate 2 or 3 times during incubation by inverting the tube.
  - Assemble a Filter Column with one of the provided collection tubes. Pipette the lysate into Filter Column and spin for 2 min at 20,000× *g*.
  - Transfer only the clear supernatant from the flowthrough into an RNase-free microcentrifuge tube.
  - Add an equal volume of 96–100% ethanol to the collected lysate. Vortex to mix.
2. Binding to Column
  - Assemble a Spin Column with one of the provided collection tubes.
  - Apply up to 600 μL of the clarified lysate with ethanol onto the column and centrifuge for 1 min at ≥3500× *g*. Discard the flowthrough and reassemble the spin column with the collection tube.
  - Depending on the lysate volume, repeat step 2b if necessary.
3. Column Wash
  - Apply 400 μL of Wash Solution A to the column and centrifuge for 1 min at 20,000× *g*.
  - Discard the flowthrough and reassemble the spin column with its collection tube.
  - Repeat steps 3a and 3b to wash the column a second time.
  - Wash the column a third time by adding another 400 μL of Wash Solution A and centrifuging for 1 min.
  - Discard the flowthrough and reassemble the spin column with its collection tube.
  - Spin the column for 2 min at 20,000× *g*. Discard the collection tube.
4. RNA Elution
  - Place the column into a fresh Elution tube.
  - Add 50 μL of Elution Solution A to the column.
  - Centrifuge for 2 min at 200× *g*, followed by a 2 min spin at 20,000× *g*.

#### 4.1.4. RNA Quality and Quantity Control

1. Measure RNA concentration using a fluorometer (Qubit).
2. Assess RNA integrity using a bioanalyzer.
3. Evaluate purity ratios (A260/A280 and A260/A230) using a spectrophotometer (Nanodrop).

Notes

- Multiple sorbitol pre-washes were tested, but the results showed no significant improvements.
- The berry skin RNA extraction was performed on multiple samples to validate the method.
- The commercial kit-based extraction was extended to additional grapevine tissues (rachis, buds, and roots) for comparison.
- A graphical protocol is shown in Supplementary Figure S2.

### 4.2. qPCR Validation

cDNA was synthesized using the qScript™ cDNA Synthesis kit (Quantabio, Beverly, MA, USA) from 1 μg of total RNA extracted from grape berry skin with and without sorbitol. Different amounts of cDNA (20 ng, 30 ng, 50 ng, and 100 ng) were used for the qPCR of housekeeping genes (ACTN and GAPC). A total of 20 ng of cDNA was used to compare the detection of genes with different expression levels (ACTN, Vitvi18g02133, and Vitvi03g00231). All the specific primers are listed in Supplementary Table S5. The qPCR reaction was conducted on a Step One Plus System (Applied Biosystem, Waltham, MA, USA) with the following cycler conditions: Holding stage—95 °C for 12 min; Cycling stage (40 times)—95 °C for 3 s and 60 °C for 30 s; and Mel curve stage—95 °C for 15 s and 60 °C for 1 min.

### 4.3. Library Preparation and RNA Sequencing

The RNA samples extracted from berries skin were redirected to the Novogene company (Cambridge, UK) for RNA-seq analysis. A total of 33 RNA-seq libraries were constructed using the Eukaryotic mRNA-Seq library (polyA enrichment, non-directional library). The RNA library was constructed using polyA capture and reverse transcription of cDNA. Library QC was performed. For each sample, the transcriptome sequencing was carried out using Illumina NovaSeq X PE150 technology (Illumina, San Diego, CA, USA).

### 4.4. Statistical Analysis

The data were analyzed using RStudio software (v. 4.2.3) and the ggplot2 package. To assess the statistical significance of differences in RNA concentrations between treatment groups, we performed a *t*-test.

## Figures and Tables

**Table 1:** Mean purity and yield values of kit-based extracted RNA with and without sorbitol pre-wash from four grape tissues.

Tissue Type	# of Samples	Mean RNA Yield ^a^		Mean A260/A280 ^b^		Mean A260/A230 ^b^		Mean RIN ^c^	
		Without Sorbitol	With Sorbitol	Without Sorbitol	With Sorbitol	Without Sorbitol	With Sorbitol	Without Sorbitol	With Sorbitol
Rachises	5	22.1 ± 11.5	6.4 ± 1.5	2.1 ± 0.1	2.1 ± 0.1	0.4 ± 0.2	0.1 ± 0	5.8 ± 1.9	0.9 ± 1.3
Buds	5	28.5 ± 10.5	36.5 ± 5.6	2.1 ± 0	2.1 ± 0	0.6 ± 0.2	0.7 ± 0.1	6.4 ± 2.3	4.8 ± 1.1
Skins	5	3.3 ± 3.4	20.8 ± 4	2.0 ± 0.3	2.1 ± 0.1	0.1 ± 0	1.2 ± 0.3	1.2 ± 1.6	7.2 ± 0.2
Roots	5	16.1 ± 15	9.3 ± 7.6	1.8 ± 0.3	2.0 ± 0.1	0.4 ± 0.2	0.2 ± 0.1	4.9 ± 3	2.8 ± 2.8

^a^ ng/µL measured with Qubit fluorimeter; ^b^ measured using NanoDrop 2000 spectrophotometer (Thermo Fisher Scientific, Waltham, MA, USA); ^c^ measured using Agilent 2100 Bioanalyzer (Agilent Technologies, Inc., Santa Clara, CA, USA).

## Data Availability

All data generated or analyzed during this study are included in the published article.

## Supplementary Materials

The following supporting information can be downloaded at: https://www.mdpi.com/article/10.3390/plants14070988/s1, Figure S1: Quantity and quality assessment of RNA extraction without sorbitol pre-wash. A. Quality and quantity of RNA extracted from berry skin without sorbitol pre-wash using a commercial kit. Left panel shows the total yield, and 260/280 and 260/230 ratios; right panel shows the bioanalyzer profile and the RIN of the sample. B. Quality and quantity of RNA extracted from berry skin without sorbitol pre-wash using a non-commercial kit. Left panel shows the total yield, and 260/280 and 260/230 ratios; right panel shows the bioanalyzer profile and the RIN of the sample. C. Quality and quantity of RNA extracted from berry skin with sorbitol pre-wash using a commercial kit. Left panel shows the total yield, and 260/280 and 260/230 ratios; right panel shows the bioanalyzer profile and the RIN of the sample. D. Quality and quantity of RNA extracted from berry skin with sorbitol pre-wash using a non-commercial kit. Left panel shows the total yield, and 260/280 and 260/230 ratios; right panel shows the bioanalyzer profile and the RIN of the sample; Figure S2: Overview of protocols used in this study. Main steps and timing of the RNA extraction methods used, comparing non-commercial and commercial kits, both with and without a sorbitol pre-wash. The non-commercial protocol (left panel) spans three days, involving lysis, washing, precipitation, and elution, while the commercial Norgen kit protocol is completed in approximately one hour, including lysis, filtration, washing, and elution. Quality and quantity control of the extracted RNA is performed using Qubit fluorometry, Nanodrop spectrophotometry, and bioanalyzer assessment; Figure S3: Effect of sorbitol pre-wash on qPCR sensitivity. Ct values of two housekeeping genes (ACTN7 and GAPC2) from RNA extracted with and without the sorbitol pre-wash using the commercial kit and different cDNA input amounts; Figure S4: Impact of sorbitol pre-wash on qPCR amplification and specificity. A. Amplification plot and melting curve of the housekeeping gene using the sample extracted with (light blue) and without sorbitol (orange) using the commercial kit. B. Amplification plot and melting curve of the moderately expressed genes using the sample extracted with (light blue) and without sorbitol (orange) using the commercial kit. C. Amplification plot and melting curve of the low-expression gene using the sample extracted with (light blue) and without sorbitol (orange) using the commercial kit; Supplementary Table S1: Purity and yield of extracted RNA, with and without sorbitol pre-wash, using the commercial kit from five grape tissues; Supplementary Table S2: Purity and yield of RNA extracted from 38 berry skin samples, with sorbitol pre-wash, using the commercial kit; Supplementary Table S3: Basic statistics of the 33 sequenced berry skin samples. The table reports the number of raw reads and clean reads after quality filtering, the error rate, and the sequencing quality scores (Q20 and Q30). Q20 and Q30 represent the percentage of bases with a Phred quality score of at least 20 and 30, respectively, indicating the base call accuracy. The GC content (%) of each sample is also provided; Supplementary Table S4: Gene expression level for each gene and in each sample, derived from the calculation of FPKM (Fragments Per Kilobase of transcript sequence per Millions base pairs sequenced) based on the abundance of transcripts (reads count), gene length, and sequencing depth. FPKM is the most common method of estimating gene expression levels, which takes the effects of both sequencing depth and gene length on counting of fragments into consideration; Supplementary Table S5: List of primers used in qPCR experiments.
