# Supplementary material for: Optimized Protocol for High-Quality RNA Extraction from Grape Berry Skins Using Sorbitol Pre-Wash
Source: Plants (Basel). 2025 Mar 21;14(7):988. doi: 10.3390/plants14070988 (PMC11990153; doi:10.3390/plants14070988)

A

Commercial Kit without sorbitol pre-wash

Sample ID	260/280	260/230	ng/ul (Qubit)
Sample_1	1.98	0.15	8.90

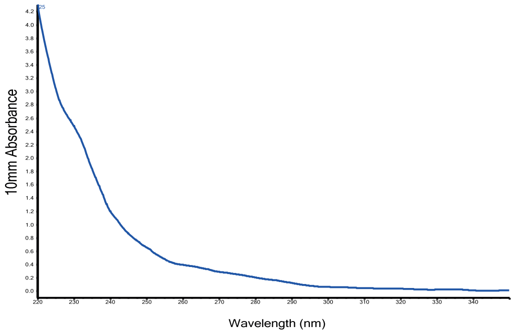

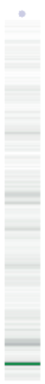

Bioanalyzer_2100 expert_Plant RNA Nano

RNAIntegrityNumber(RIN):	3.5

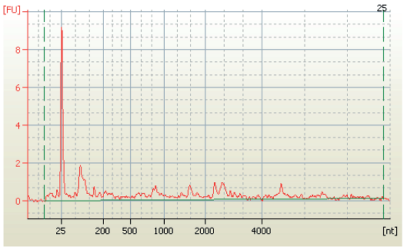

B

Non-commercial Kit without sorbitol pre-wash

Sample ID	260/280	260/230	ng/ul (Qubit)
Sample_1	1.31	0.30	4

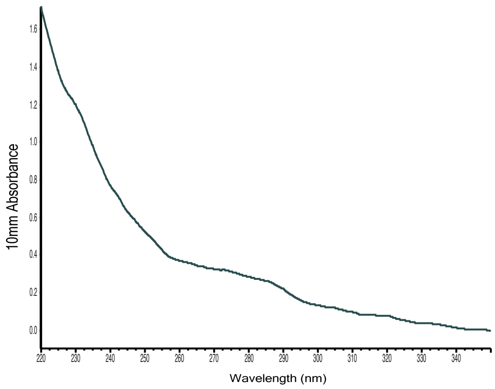

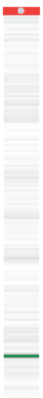

Bioanalyzer_2100 expert_Plant RNA Nano

RNAIntegrityNumber(RIN):	N/A

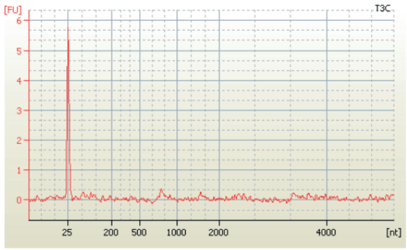

C

Commercial Kit with sorbitol pre-wash

Sample ID	260/280	260/230	ng/ul (Qubit)
Sample_1	2.15	1.46	23.71

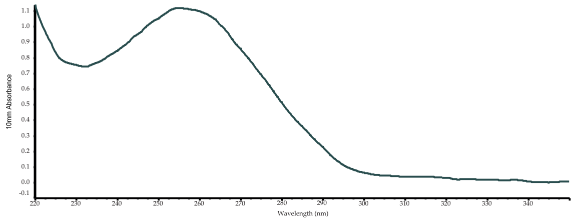

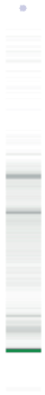

Bioanalyzer_2100 expert_Plant RNA Nano

RNAIntegrityNumber(RIN):	7.2

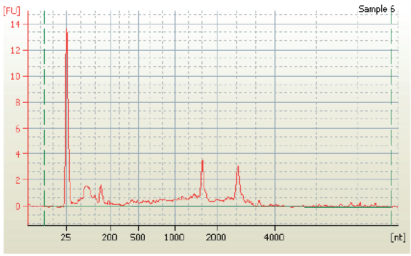

D

Non-commercial Kit with sorbitol pre-wash

Sample ID	260/280	260/230	ng/ul (Qubit)
Sample_1	1.81	1.15	16.24

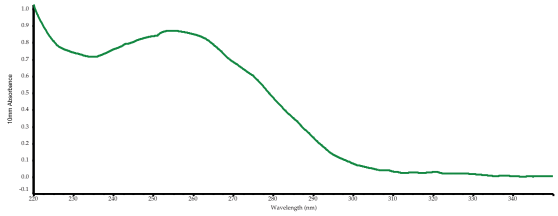

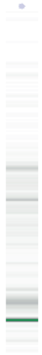

Bioanalyzer_2100 expert_Plant RNA Nano

RNAIntegrityNumber(RIN):	7.6

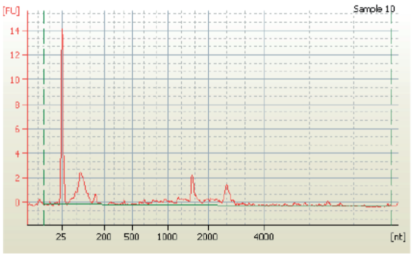

Supplement: Supplementary file 1 [file plants-14-00988-s001.zip › Prencipe_Supplementary/Prencipe_FigureS1.pdf]
