# Supplementary material for: Optimized Protocol for High-Quality RNA Extraction from Grape Berry Skins Using Sorbitol Pre-Wash
Source: Plants (Basel). 2025 Mar 21;14(7):988. doi: 10.3390/plants14070988 (PMC11990153; doi:10.3390/plants14070988)
Supplement: Supplementary file 1 [file plants-14-00988-s001.zip › Prencipe_Supplementary/Prencipe_FigureS2.pdf]

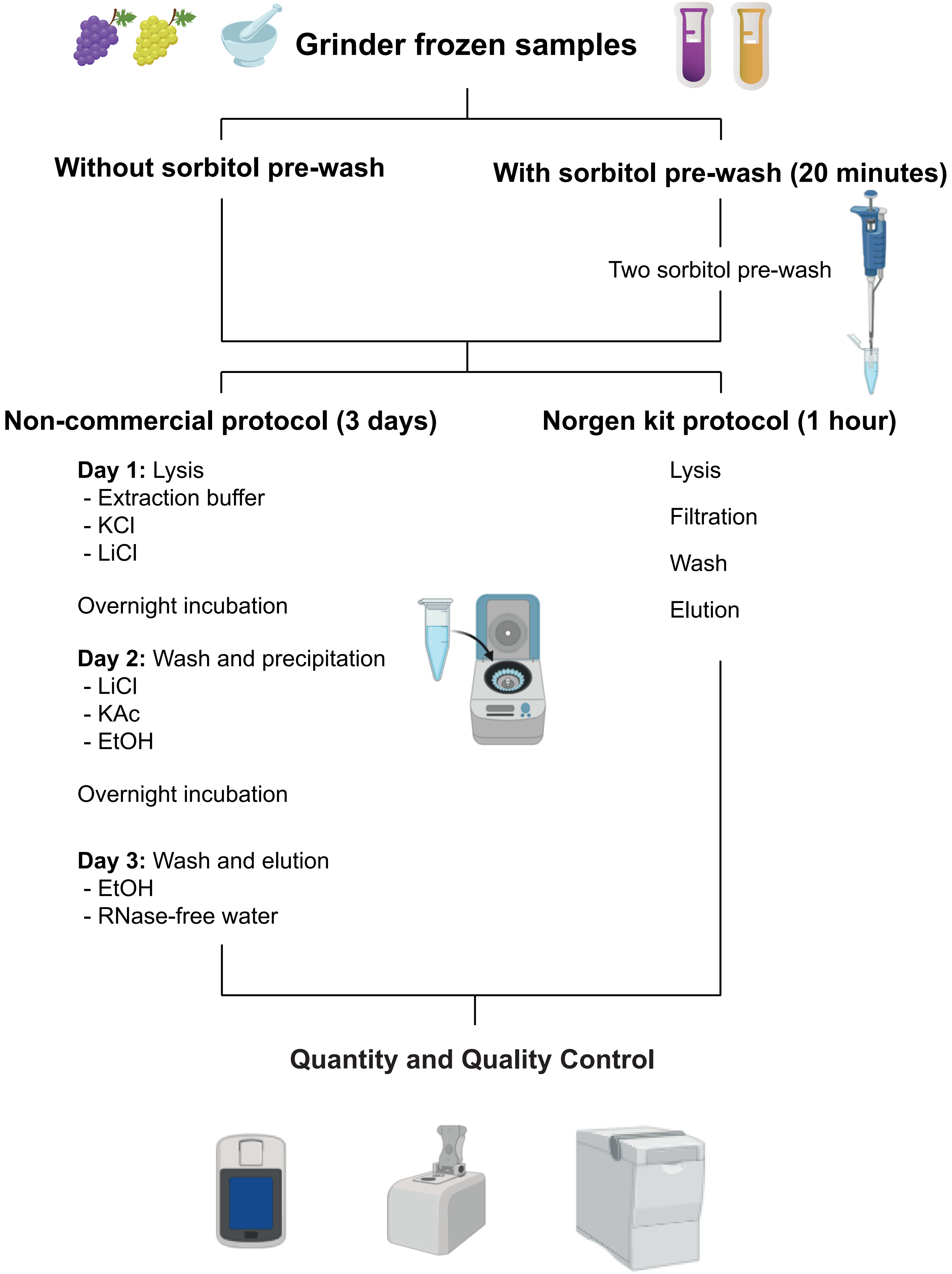

Grinder frozen samples
Without sorbitol pre-wash
With sorbitol pre-wash (20 minutes)
Two sorbitol pre-wash
Non-commercial protocol (3 days)
Day 1: Lysis
- Extraction buffer
- KCl
- LiCl
Overnight incubation
Day 2: Wash and precipitation
- LiCl
- KAc
- EtOH
Overnight incubation
Day 3: Wash and elution
- EtOH
- RNase-free water
Norgen kit protocol (1 hour)
Lysis
Filtration
Wash
Elution
Quantity and Quality Control
