# Supplementary figures and images for: Optimized Protocol for High-Quality RNA Extraction from Grape Berry Skins Using Sorbitol Pre-Wash
Source: Plants (Basel). 2025 Mar 21;14(7):988. doi: 10.3390/plants14070988 (PMC11990153; doi:10.3390/plants14070988)

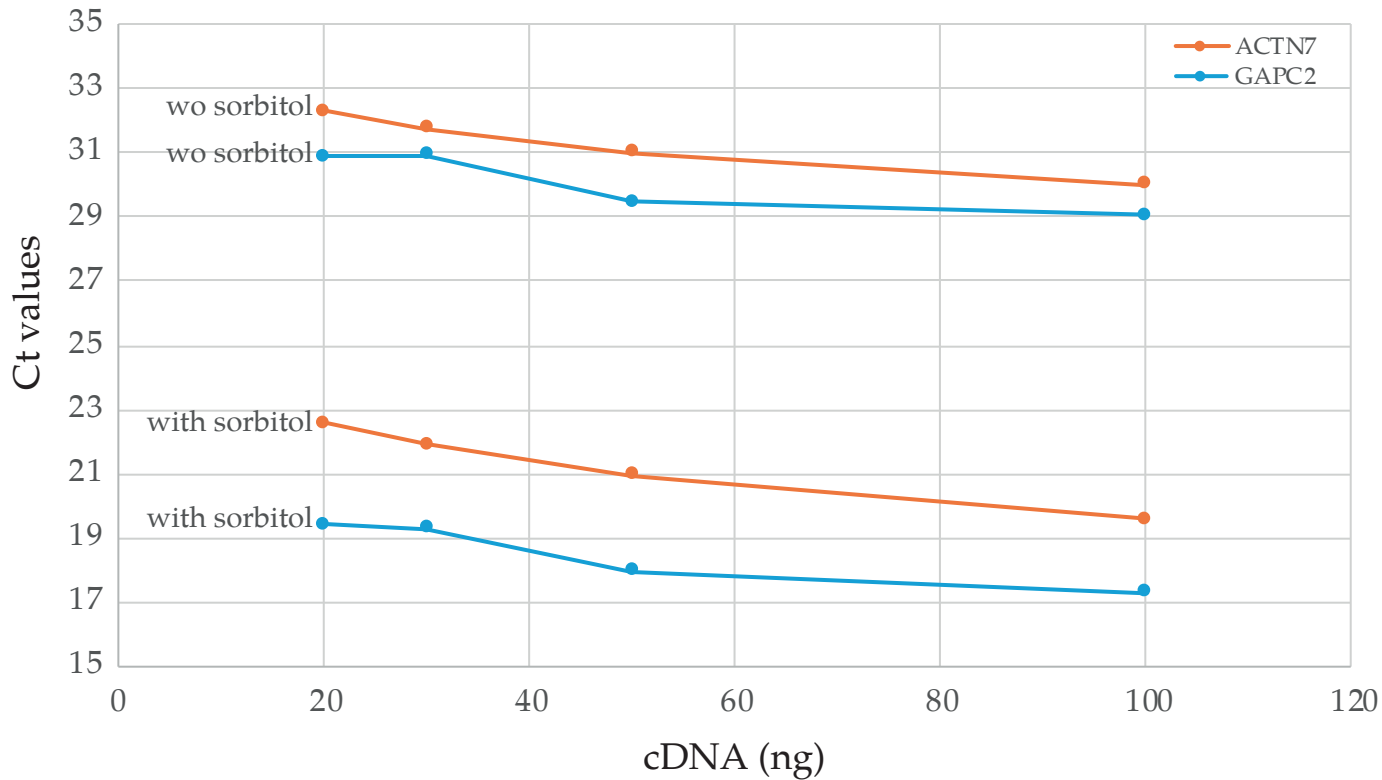

Supplement: Supplementary file 1 [file plants-14-00988-s001.zip › Prencipe_Supplementary/Prencipe_FigureS3.pdf]

A

Housekeeping gene

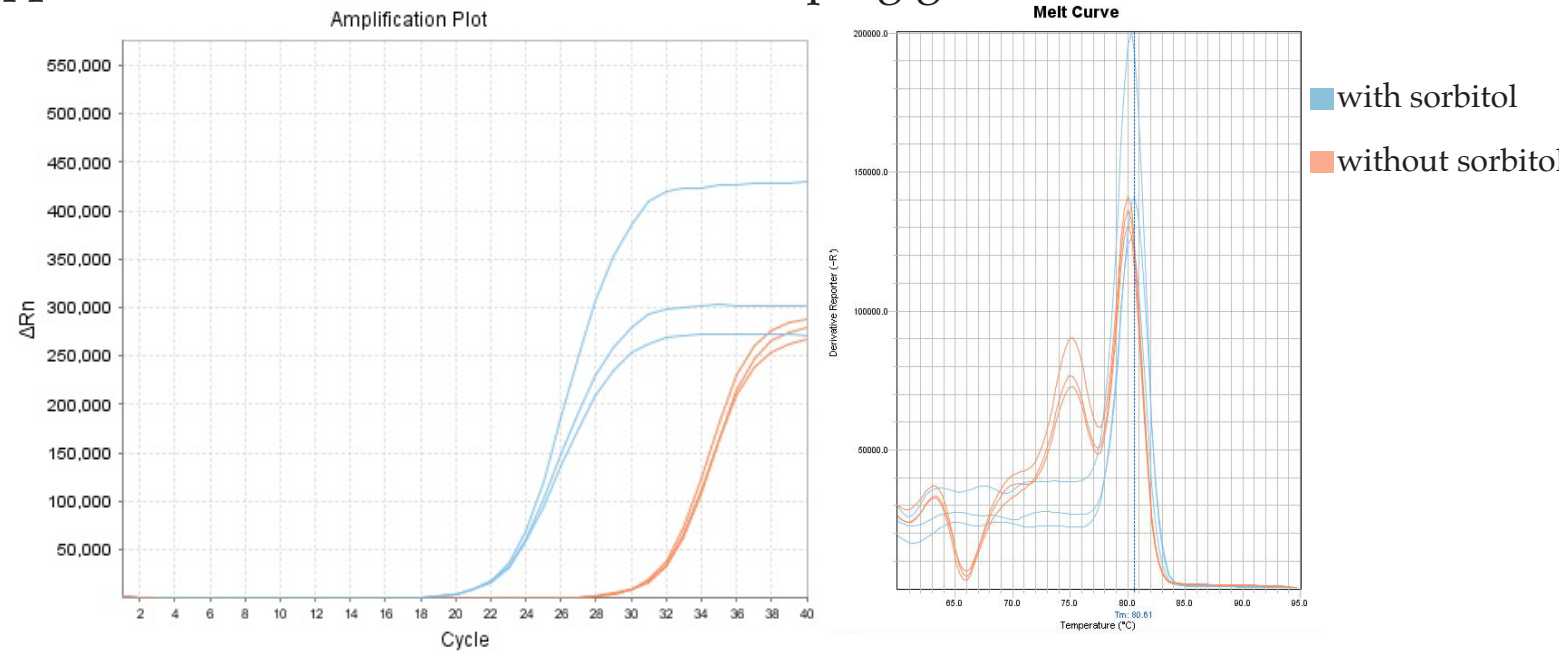

B

Medium-expressed gene

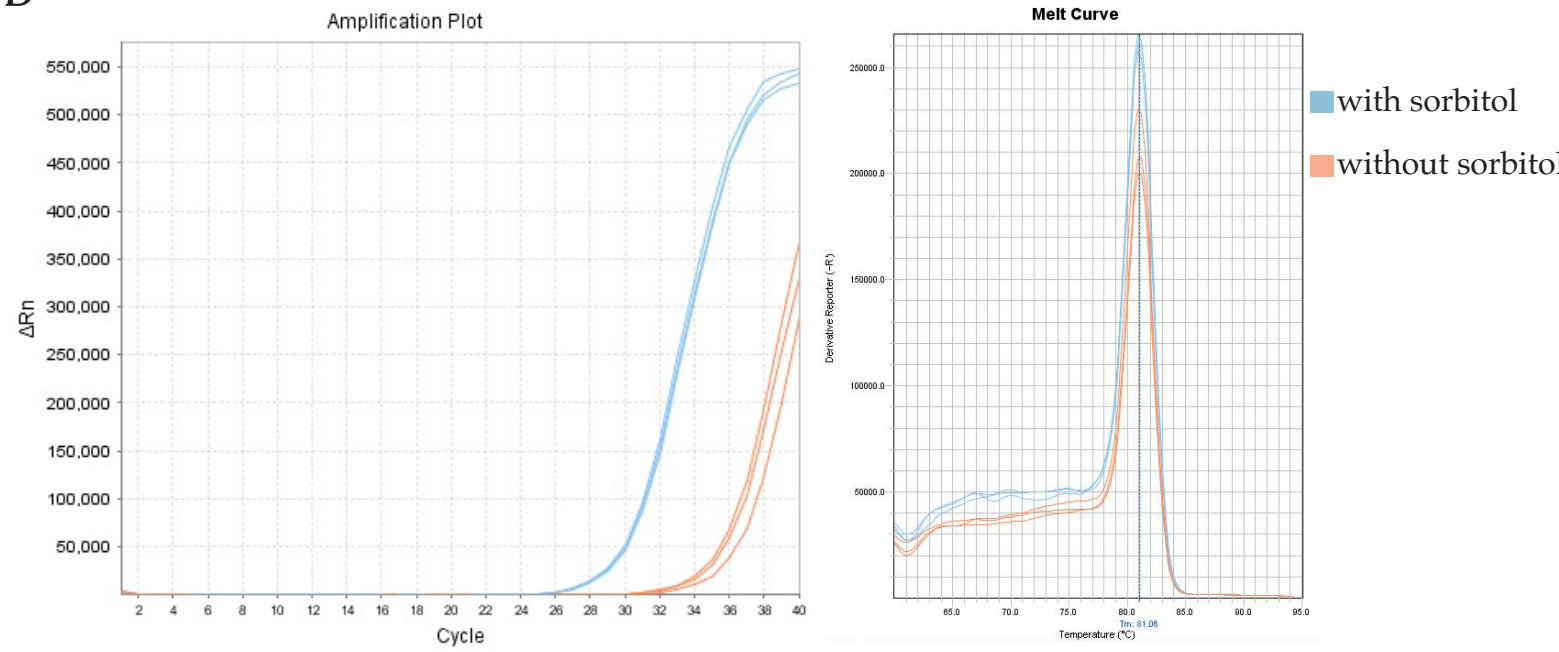

C

Low-expressed gene

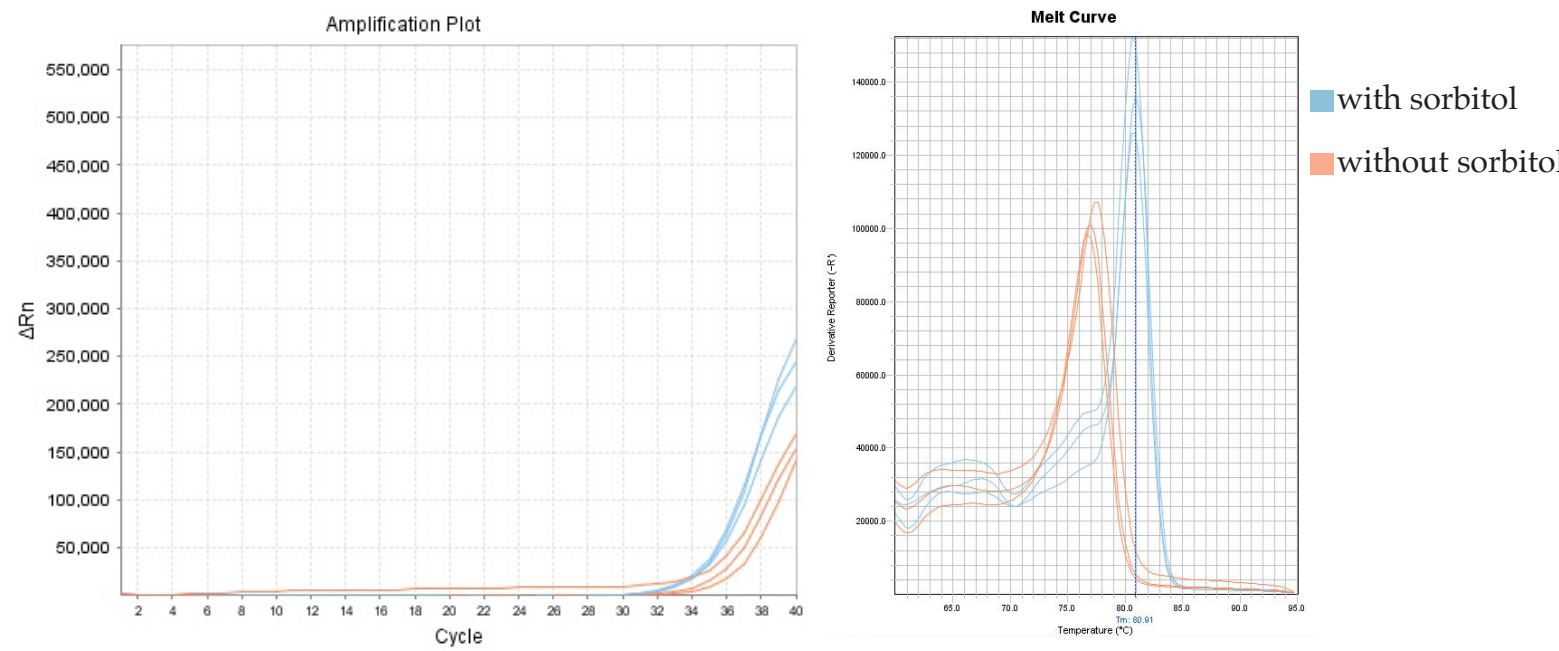

Supplement: Supplementary file 1 [file plants-14-00988-s001.zip › Prencipe_Supplementary/Prencipe_FigureS4.pdf]
